# Nirmatrelvir/Ritonavir for hemodialysis patients with COVID-19

**DOI:** 10.3389/fphar.2023.1161897

**Published:** 2023-05-12

**Authors:** Jiayue Lu, Hong Cai, Yujun Hao, Zhang Lin, Shang Liu, Yaping Zhan, Li Ding, Meilan Huang, Zhenyuan Li, Lan Xu, Xiujuan Yan, Li Yang, He Zhang, Wei Zhang, Li Zhao, Junli Zhao, Ting Wang, Leyi Gu

**Affiliations:** ^1^ Department of Nephrology, Molecular Cell Lab for Kidney Disease, Shanghai Peritoneal Dialysis Research Center, Renji Hospital, Uremia Diagnosis and Treatment Center, Shanghai Jiaotong University, School of Medicine, Shanghai, China; ^2^ State Key Laboratory of Oncogenes and Related Genes, Shanghai Cancer Institute, Renji Hospital, Shanghai Jiao Tong University School of Medicine, Shanghai, China; ^3^ Department of Gastroenterology, Renji Hospital, School of Medicine, Shanghai Jiaotong University, Shanghai, China; ^4^ Department of Hematology, Renji Hospital, School of Medicine, Shanghai Jiaotong University, Shanghai, China; ^5^ Department of Nephrology, Zhoupu Hospital, Shanghai University of Medicine and Health Sciences, Shanghai, China

**Keywords:** hemodialysis, nirmatrelvir, ritonavir, pharmacokinetics, COVID-19

## Abstract

**Background:** Hemodialysis patients have a high risk of severe/critical COVID-19 and related high mortality, but nirmatrelvir/ritonavir is not recommended for hemodialysis patients with COVID-19 infection because of lack of evidence of safety.

**Objectives:** Our study aims to evaluate the minimum plasma concentration (Cmin) of nirmatrelvir and its safety of different doses of nirmatrelvir/ritonavir in hemodialysis patients with mild COVID-19.

**Method:** This was a prospective, two step, nonrandomized, open-label study. Participants were treated with nirmatrelvir 150 mg or 300 mg once a day (another 75 mg or 150 mg supplied after hemodialysis) and ritonavir 100 mg twice daily for 5 days, respectively. The primary outcome was the safety of nirmatrelvir/ritonavir, including the Cmin of nirmatrelvir and the number of adverse events (AE). The secondary outcome was the time of viral elimination in hemodialysis patients.

**Results:** Adverse events were happened in 3 and 7 participants in the step 1 and step 2 group, respectively (*p* = 0.025). Among them, 2 and 6 participants were identified as drug-related adverse events (*p* = 0.054). No SAE or liver function damage happened. The Cmin of nirmatrelvir in step 1 and step 2 group were 5,294.65 ± 2,370.59 ng/mL and 7,675.67 ± 2,745.22 ng/mL (*p* = 0.125). The Cmin of the control group was 2,274.10 ± 1,347.25 ng/mL (*p* = 0.001 compared to step 2 and *p* = 0.059 compared to step 1). Compared to hemodialysis patients without nirmatrelvir/ritonavir, there were no statistical differences in overall viral elimination time (*p* = 0.232).

**Conclusion:** In our study, two doses of nirmatrelvir/ritonavir appeared to be excessive for hemodialysis patients. Although all of the patients tolerated 5-day administration, nearly half of the patients experienced drug-related adverse events. In addition, the medication group did not show a significant advantage in the time of viral elimination.

## Introduction

In the last 3 years, COVID-19 has threatened the health of the global population. People over 60 years old, smoking or suffering from cardiovascular disease, obesity, immune deficiency, liver disease, and chronic kidney disease have a higher risk of severe/critical COVID-19 and related high mortality (Zhang et al., 2020; Terada et al., 2021; Western Cape Department of Health in collaboration with the National Institute for Communicable Diseases and South Africa, 2021). The incidence rate and mortality of COVID-19 in patients receiving maintenance dialysis were higher than those in patients with chronic kidney disease who did not need renal replacement therapy (Hsu et al., 2021). It had been demonstrated that treatment with nirmatrelvir/ritonavir reduce the risk of progression to severe COVID-19. The World Health Organization strongly recommended the use of nimatrivir/ritonavir for patients with a high risk of hospitalization, as it can effectively reduce the risk of developing serious diseases (Agarwal et al., 2020). However, due to the lack of clinical trial data, nimatrivir/ritonavir has not been recommended for hemodialysis patients with COVID-19 infection. In this study, we conducted a prospective study to evaluate the minimum plasma concentration (Cmin) of nirmatravir and its safety of different doses of nirmatravir/ritonavir in hemodialysis patients with mild COVID-19, as well as its effect in this special population.

## Methods

### Design and participants

This was a prospective, two step, nonrandomized, open-label study, and was conducted from April 2022 to June 2022. Eligible patients were between 18 and 75 years of age, who had received hemodialysis twice or three times a week for more than 1 month with COVID-19 infection. The complete inclusion/exclusion criteria are provided in the Supplementary File. Six patients, who had normal renal function with mild COVID-19 infection were used as a reference for the plasma concentration of nirmatrelvir. Thirty-five hemodialysis patients without nirmatrelvir/ritonavir treatment were compared for the viral elimination time. The study was carried out in accordance with the Declaration of Helsinki. All patients provided their written informed consent prior to screening. The study protocol and the informed consent form were approved by the Ethics Committee of Renji Hospital. The Clinical Trial registration number was NCT05366192.

### Treatment and outcomes

In the step 1 group, participants were treated with nirmatrelvir 150 mg once daily (75 mg supplied after hemodialysis) and ritonavir 100 mg twice daily for 5 days. In the step 2 group, participants were treated with nirmatrelvir 300 mg once daily (150 mg supplied after hemodialysis) and ritonavir 100 mg twice daily for 5 days. In the control group, participants were treated with nirmatrelvir 300 mg twice daily and ritonavir 100 mg twice daily for 5 days. All of the hemodialysis patients received hemodialysis treatment on day 2 and day 4. No hemodiafiltration and hemoperfusion were performed during the study. Routine blood samples were collected at 8 a.m. on day 0, day 3 and day 6, including routine blood test, C-reactive protein, creatine kinase, D-Dimer and liver function. The concentration of nirmatrelvir was collected at 8 a.m. on day 6. Nasal swab were collected every morning from day 1 to day 10 or until viral load less than 500 copies/mL.

The primary outcome was the safety of nirmatrelvir/ritonavir, including the Cmin of nirmatrelvir and the incidence of adverse events(AE). Adverse events included: the number of patients with adverse events during day 1 to day 14 and the number of patients with deterioration of liver function (ALT, AST, total bilirubin, and direct biirubin increase to 2 times from baseline) on day 3 and day 6. AE were recorded by doctors during daily rounds. The secondary outcome was the time of viral elimination in hemodialysis patients, from the first day of hospitalization to viral elimination. Viral elimination was defined as both negative for ORF1ab and N genes (Ct value ≥ 35 by PCR) on 2 consecutive days.

### Statistical methods

The normality of distribution of continuous variables was tested by Shapiro–Wilk test. Continuous variables with normal distribution were presented as mean (standard deviation [SD]); non-normal variables were reported as median (interquartile range [IQR]). Categorical variables were expressed as constituent ratios or percentages. The comparisons of constituent ratio between 2 steps by using Fisher’s exact test. 2-sample Poisson rate test was used to determine if there was a significant difference between the adverse event rates of occurrence of two groups. Repeated measure ANOVA or Friedman test were conducted for overtime laboratory results within groups, pairwise *t*-test with Bonferroni adjustment or Nemenyi *post hoc* test were performed for multiple comparisons. T-test or Mann-Whitney U test were used to compare the data between 2 groups. Kaplan-Meier analysis was used to compare the time of virus elimination. Data analysis was carried out with SPSS 23.0 and R 4.0.3, and *p* < 0.05 was considered statistically significant.

## Results

20 hemodialysis patients with COVID-19 were screened and 18 were eligible. 14 patients were infected for the first time and 4 patients were infected for the second time. Ten participants were in the step 1 group and eight were in the step 2 group. Both groups were balanced with demographics, baseline disease characteristics and hemodialysis status (Table 1).

**TABLE 1:** Demographic and clinical characteristics of the patients.

Characteristic	Total patients (N = 18)	Step 1 group (N = 10)	Step 2 group (N = 8)	*P* (step1 vs. step 2)
Age (years)	71.00 (57.00–76.00)	75.00 (63.75–77.25)	62.00 (56.00–72.25)	0.247
Female, n (%)	8 (44)	4 (40)	4 (50)	1.000
Primary cause for Hemodialysis, n (%)	5 (28)	4 (40)	1 (13)	0.314
Chronic glomerulonephritis	7 (39)	3 (30)	4 (50)	0.630
Diabetes kidney disease	3 (17)	1 (10)	2 (25)	0.559
Hypertension	1 (6)	1 (10)	0	1.000
ADPKD	2 (11)	1 (10)	1 (13)	1.000
Unknown causes				
Complications, n (%)	8 (44)	3 (30)	5 (63)	0.342
Diabetes	4 (22)	3 (30)	1 (13)	0.588
Coronary heart disease	2 (11)	2 (20)	0	0.477
Bacterial pneumonia	2 (11)	0	2 (25)	0.183
Cerebral infarction	1 (6)	0	1 (13)	0.444
Renal transplantation	1 (6)	1 (10)	0	1.000
Intestinal cancer	1 (6)	1 (10)	0	1.000
Renal hemorrhage	1 (6)	1 (10)	0	1.000
COPD				
Hemodialysis vintage (months)	17 (3.75–93.00)	27 (3.00–120.00)	14 (5.25–72.00)	0.562
AV fistula, n (%)	10 (56)	6 (60)	4 (50)	1.000
Kt/V	1.13 ± 0.23	1.12 ± 0.23	1.15 ± 0.24	0.845
URR (%)	62.33 ± 8.40	62.26 ± 8.46	62.43 ± 8.96	0.969
Time since first virus positive (days)	7 (2.00–9.75)	6.5 (2.00–7.00)	10 (2.25–27.25)	0.126
Vaccine, n (%)	1 (6)	1 (10)	0 (0)	1.000
CT value of SARS-CoV-2 ORF1ab gene in PCR detection	27.51 ± 7.62	25.06 ± 7.08	30.58 ± 7.56	0.130
CT value of SARS-CoV-2 N gene in PCR detection	26.94 ± 6.35	24.77 ± 5.68	29.65 ± 6.44	0.107

**FIGURE 1:**
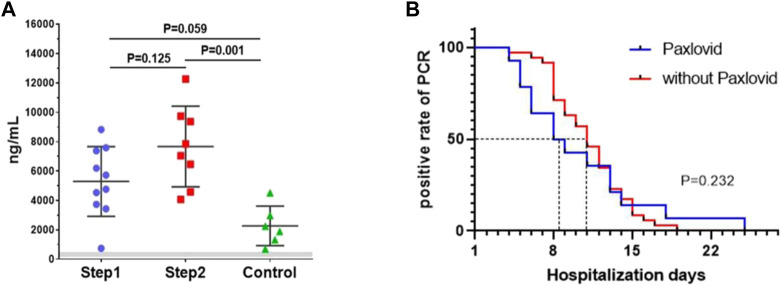
**(A)** The Cmin of three groups (step 1 group, step 2 group, control group). Bar showed means ± sd. Gray zone is *in vitro* 90% effective concentration of 124–685 ng/mL (account for plasma protein binding); **(B)**. *in vitro* Kaplan-Meier analysis of the viral elimination time.

As a result, adverse events were happened in 3 and 7 participants in the step 1 and step 2 group, respectively (*p* = 0.025). Among them, 2 and 6 participants were identified as drug-related adverse events (*p* = 0.054). The number of adverse events were 4 and 11 respectively (*p* = 0.046). Gastrointestinal discomfort was the most common AE. No SAE occurred and no participant had a double of ALT, AST, total bilirubin, direct bilirubin from baseline in both groups. White blood cells, lymphocyte count, C-reactive protein, creatine kinase, and d-dimer did not show significant changes in both groups (Table 2). All of the patients tolerated 5-day administration. The Cmin of nirmatrelvir in the step 1 and step 2 group were 5,294.65 ± 2,370.59 ng/mL, 7,675.67 ± 2,745.22 ng/mL (*p* = 0.125), respectively. The Cmin of the control group was 2,274.10 ± 1,347.25 ng/mL (*p* = 0.001 compared to step 2 and *p* = 0.059 compared to step 1) (Figure 1A). After excluding patients with reinfection, the median viral elimination time was shorter in the treated group compared to hemodialysis patients without nirmatrelvir/ritonavir (14 vs. 35 hemodialysis patients, 8.5 vs. 11 days), but there were no statistical differences in overall viral elimination time (*p* = 0.232) (Figure 1B). The baseline features of two groups are shown in supplementary file, Table 1.

**TABLE 2:** Adverse events until 14 days and Laboratory results.

	Total patients (N = 18)	Step 1 group (N = 10)	Step 2 group (N = 8)	*P* (step1 vs. step2)
Patients with deterioration of liver function during treatment	0	0	0	1.000
Patients with any AE, n (%)	10 (56)	3 (30)	7 (88)	0.025
Considered to be related to drugs	8 (44)	2 (20)	6 (75)	0.054
Number of any AE	15	4	11	0.046
Considered to be related to drugs	12	3	9	0.065
Grade of AE, n (%)	9 (60)	3(75)	6 (55)	0.604
1	6 (40)	1 (25)	5 (45)	
2				
Type of AEs	12	3	9	0.065
Gastrointestinal discomforts	5	2	5	1.000
Decreased appetite	3	1	2	1.000
Diarrhea	2	0	2	
Constipation	1	0	1	
Abdominal pain	2	1	1	
Chest distress				
Laboratory results	5.85 ± 3.35	6.75 ± 3.66	4.72 ± 2.73	0.211
WBC (×10^9^/L)				
Day0				
Day3	6.00 ± 2.97	6.51 ± 2.56	5.36 ± 3.48	0.430
Day6	6.56 ± 2.53	6.80 ± 2.12	6.26 ± 3.10	0.666
*P* (D0 vs. D3 vs. D6)	0.394	0.877	0.314	
Lymphocyte (×10^9^/L)				
Day0	0.84 ± 0.38	0.86 ± 0.23	0.82 ± 0.53	0.812
Day3	0.92 ± 0.31	0.86 ± 0.20	0.99 ± 0.41	0.391
Day6	0.99 ± 0.33	0.92 ± 0.27	1.07 ± 0.39	0.353
*P* (D0 vs. D3 vs. D6)	0.347	0.748	0.437	
Hemoglobin (g/L)				
Day0	93.78 ± 18.30	100.90 ± 18.00	84.88 ± 15.29	0.062
Day3	91.39 ± 21.05	98.80 ± 23.03	82.13 ± 14.75	0.095
Day6	86.83 ± 17.81	92.10 ± 18.06	80.25 ± 16.19	0.167
*P* (D0 vs. D3 vs. D6)	0.006	0.020	0.288	
Platelet (×10^9^/L)	162.50 (99.25–232.25)	162.50 (104.75–243.50)	151.00 (103.25–220.75)	0.920
Day0	147.00 (100.00–250.50)	158.50 (102.75–257.50)	132.00 (103.00–174.00)	0.657
Day3	194.00 (126.75–223.75)	194.00 (143.50–218.00)	188.00 (118.00–231.75)	0.859
Day6	0.013	0.565	0.003	
*P* (D0 vs. D3 vs. D6)				
CRP (mg/L)	10.92 (3.5–45.16)	13.70 (6.43–64.94)	6.77 (2.16–39.86)	0.248
Day0	17.85 (4.40–41.07)	22.92 (7.86–54.00)	6.74 (3.17–40.13)	0.248
Day3	12.49 (5.12–55.08)	21.08 (10.75–65.18)	7.64 (3.05–37.86)	0.155
Day6	0.624	0.888	0.860	
*P* (D0 vs. D3 vs. D6)				
Alt (U/L)	15.00 (8.00–27.25)	26.00 (11.00–38.50)	13.5 (7.50–16.00)	0.090
Day0	13.50 (6.5–19.25)	19.00 (10.25–24.50)	11.0 (6.50–14.25)	0.082
Day3	11.00 (7.50–9.75)	11.00 (8.50–12.75)	11.0 (8.25–13.50) 0.240	0.964
Day6	0.042	0.063		
*P* (D0 vs. D3 vs. D6)				
Ast(U/L)	22.44 ± 8.21	25.40 ± 8.73	18.75 ± 6.11	0.087
Day0				
Day3	18.89 ± 7.39	20.30 ± 6.99	17.13 ± 7.95	0.381
Day6	16.00 ± 5.70	15.30 ± 6.57	16.88 ± 4.67	0.576
*P* (D0 vs. D3 vs. D6)	0.000	0.000	0.364	
Total bilirubin (μmol/L)				
Day0	9.60 ± 3.88	10.93 ± 4.82	7.94 ± 0.96	0.105
Day3	10.05 ± 4.87	14.41 ± 6.08	8.35 ± 2.05	0.194
Day6	8.67 ± 3.12	9.54 ± 3.89	7.58 ± 1.30	0.192
*P* (D0 vs. D3 vs. D6)	0.016	0.049	0.330	
Direct bilirubin (μmol/L)				
Day0	1.83 ± 1.18	2.22 ± 1.47	1.34 ± 0.32	0.117
Day3	2.04 ± 1.51	2.50 ± 1.92	1.46 ± 0.29	0.152
Day6	1.75 ± 1.08	2.21 ± 1.25	1.18 ± 0.39	0.040
*P* (D0 vs. D3 vs. D6)	0.182	0.506	0.006	
Albumin (g/L)				
Day0	35.11 ± 4.82	35.36 ± 4.64	34.80 ± 5.35	0.815
Day3	33.86 ± 5.28	33.40 ± 4.84	34.44 ± 6.08	0.692
Day6	33.18 ± 5.08	32.37 ± 3.75	34.19 ± 6.51	0.467
*P* (D0 vs. D3 vs. D6)	0.031	0.009	0.865	
Creatine Kinase (U/L)	69.00 (40.00–86.50)	69.00 (38.00–110.25)	69.50 (46.75–82.25)	0.929
Day0	41.50 (30.75–70.75)	50.50 (33.00–72.25)	40.50 (28.50–54.50)	0.563
Day3	52.00 (30.5–62.50)	52.00 (35.5–57.00)	50.50 (28.75–75.25)	0.790
Day6	0.097	0.387	0.071	
*P* (D0 vs. D3 vs. D6)				
D-Dimer (DDU μg/mL)	0.37 (0.27–1.02)	0.34 (0.34–1.21)	0.45 (0.26–0.64)	0.689
Day0	0.42 (0.27–0.71)	0.50 (0.27–0.95)	0.35 (0.23–0.52)	0.328
Day3	0.46 (0.30–0.79)	0.61 (0.33–0.94)	0.37 (0.25–0.60)	0.790
Day6	0.616	0.678	0.312	
*P* (D0 vs. D3 vs. D6)				

## Discussion

In our study, the infection of COVID-19 was mainly caused by Omicron variant strains, which had stronger transmissibility and weaker pathogenicity to the general population compared to other strains. Although treatment strategies for COVID-19 are still largely supportive and prevention of complications, antiviral therapies prevents progression to severe/critical COVID-19 and promotes clinical recovery in COVID-19 infected patients. Nirmatrelvir/ritonavir was strongly recommended by WHO for patients with non-severe COVID-19 at high risk of hospitalization. Because lack of trial data, the drug is still unavailable for dialysis patients with COVID-19 infection although dialysis patients suffering COVID-19 has higher mortality.

The pharmacokinetics of nirmatrevir/ritonavir in hemodialysis patients was seldom reported before our study. Ritonavir, which acts as a pharmacokinetic enhancer, is a CYP3A4 inhibitor and enhances nirmatrelvir’s bioavailability. Since ritonavir is mostly hepatically metabolized and is 99% protein bound, the dose of ritonavir was not adjusted in our study. Nirmatrelvir has a molecular mass of 499.5 D, 35% is approximately excreted by the kidneys, and it is 70% protein bound. As reported, the AUCinf and T1/2 of nirmatrelver increases rapidly in patients with eGFR<30 mL/min. Meanwhile, hemodialysis can remove up to 31% unbinding nirmatrelvir (fda, 2021). So we tried 1/4 dose and half dose of nirmatrelvir for hemodialysis patients, and half of research dose of nirmatrelvir was supplied after dialysis. Due to the half-life of nirmatrelvir being 6 h and the concentration of nirmatrelvir rapidly decreases 24 h after administration in patients with normal renal function, we chose to measure its blood concentration 1 day after the end of medication, as the minimum plasma concentration(Cmin).

As former literature reported, the effective concentration of 90% *in vitro* (EC90, account for plasma protein binding) was 124–685 ng/mL (Owen et al., 2021). In the present study, Cmin in the step 1 group was 7.7 times higher than the up-limitation of the effective concentration *in vitro* and 2.3 times higher than the concentration in our control group (Singh et al., 2022), indicated that nirmatrelvir was overdosed and its metabolism was slow. We also noticed that the Cmin was very variable in our participants. This may be related to various factors such as the weight, nutritional status, concomitant medication, and dialysis adequacy of hemodialysis patients. Therefore, it is necessary to examine the Cmin in hemodialysis patients treated with the proposed dose of nirmatrelvir and ritonavir. Meanwhile, according to the incidence of AE in former phase 2-3 clinical trial (22.6%) (Hammond et al., 2022), the incidence of AE was high in both groups, especially in the step 2 group, with a dose-dependent effect. Gastrointestinal discomforts were the most common AE. But all of the patients finished the 5-day treatment, and there were no SAE happened, no any deterioration in laboratory results, suggesting that a lower dose of nirmatrelvir/ritonavir would be safe in hemodialysis patients. Besides, all of the patients in this study were mild COVID-19 infections, no death and no severe infection happened during hospitalization. So we can only use virus elimination time as a reference indicator for drug efficacy. Our study showed that the median viral elimination time was shorter in the treated group compared to hemodialysis patients without nirmatrelvir/ritonavir, but there were no statistical differences in overall viral elimination time. This result may be related to a small number of cases and all of the included cases were mild infection. So, the validity of the drug in hemodialysis patients requires further clinical research to verify.

This study had some limitations. First, to reduce the number of repeated blood testing in hemodialysis patients, we only measured the concentration of nirmatrelvir one time, so we cannot speculate on the impact of single hemodialysis treatment on drug clearance and plot the area under the plasma concentration-time curve during the 5-day medication. Second, before we conducted this study, there was no recommended dosage for hemodialysis patients. So we developed a dose based on the published pharmacokinetic profile of nirmatrelvir. When our study finished, Hiremath et al. (2022) proposed the recommended dosage of drugs in dialysis patients: 300 mg nirmatrelvir + 100 mg ritonavir both on day 1, then 150 mg nirmatrelvir + 100 mg ritonavir once a day for 4 more days, to be dosed after dialysis. The recommended dose is similar to the dose of step 1 in our study.

In conclusion, our doses of nirmatrelvir/ritonavir still appeared to be excessive for hemodialysis patients. Although all of the patients tolerated 5-day administration and there was no SAE or liver function damage, the Cmin of the drug was higher than that of the control group, and nearly half of the patients experienced drug-related adverse events, mainly gastrointestinal symptoms, with a dose-dependent effect. In addition, the medication group did not show a significant advantage in the time of viral elimination, maybe related to a small number of cases and weakened virus pathogenicity.

## Data Availability

The original contributions presented in the study are included in the article/Supplementary Material, further inquiries can be directed to the corresponding authors.
