# Supplementary material for: Nirmatrelvir/Ritonavir for hemodialysis patients with COVID-19
Source: Front Pharmacol. 2023 May 12;14:1161897. doi: 10.3389/fphar.2023.1161897 (PMC10213535; doi:10.3389/fphar.2023.1161897)
Supplement: Supplementary file 1 [file DataSheet1.pdf]

Supplementary file

Clinical Trial registry name and registration number: The safety of Paxlovid in hemodialysis patients with Covid-19. (NCT05366192)

Members of Data Monitoring Committee:

1. Yong Cai, PhD. School of Public Health affiliated with School of Medicine, Shanghai Jiao Tong University, Shanghai, China.
2. Bingshun Wang, PhD. Department of Biostatistics, Clinical Research Institute, Shanghai Jiao Tong University School of Medicine, Shanghai, China.
3. Zhichao Jin, PhD. Department of Health Statistics, Second Military Medical University, Shanghai 200433, China.

Design

This is a prospective, two-step study.

Study flow chart

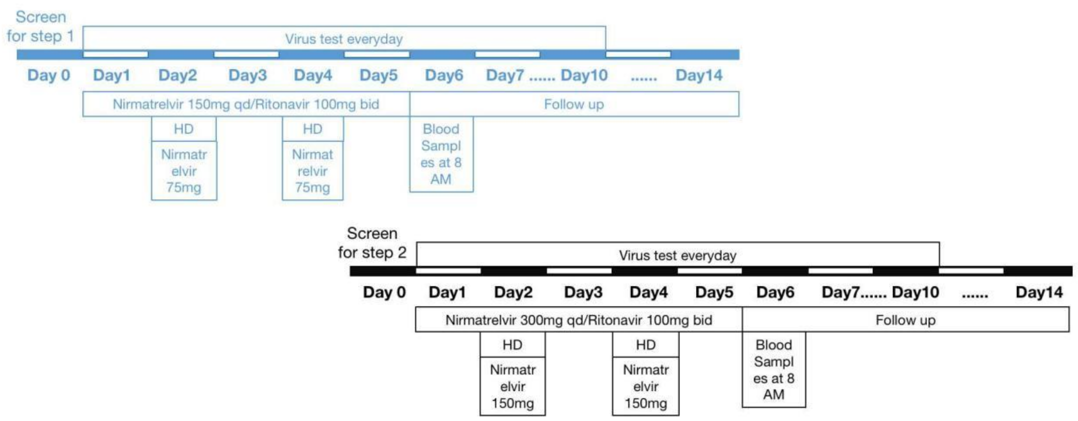

Inclusion criteria

1. Understand the present study/Agree and sign informed consent.
2. Age is between 18 and 75 at the screening
3. Blood purification twice or three times/week, including hemodialysis, hemofiltration, hemodiafiltration, CRRT or hybrid blood purification.
4. Regular hemodialysis≥1 month
5. Patients with arteriovenous fistula or artificial arteriovenous fistula.
6. Patients infected with Covid-19, the PCR CT value of nucleic acid detection<35.

Exclusion Criteria

1. Blood purification treatment<1month.
2. Liver function (ALT or AST) is three times the upper the normal.
3. The severe or critical patients with Covid-19.

4. Drug that highly dependent on CYP3A, including but not limit to afzosin, pethidine, ranolazine, amiodarone, propafenone, quinidine, fusidic acid, voriconazole, terfenadine, colchicine, rifabutin, clozapine, dihydroergot, cisapride, simvastatin, diazepam, salbutam, triazolam, carbamazepine, phenobarbital, rifampicin.
5. Patients with galactose intolerance.
6. Pregnant and lactating women.
7. Patients was allergic to paxlovid.
8. Participating in other intervention studies.
9. The investigator judged that the condition of the subjects was not suitable for the study.

SARS-CoV-2 detecting

Nasal swab collection nurses received uniform training. Samples were collected every morning from day 1 to day 10 or until viral load less than 500 copies/mL. SARS-CoV-2 gene (both ORFlab gene and N gene) detection kits (including primers, TaqMan probe and RNase P gene positive control) were purchased from Shanghai BioGerm Medical Technology Company. The low limitation of detection was 500 copies/mL. RT-PCR condition was followed manual book and performed by department of clinical laboratory in Renji Hospital.

Specimen pretreatment and LC-MS/MS assay

Blood specimens were collected in sodium heparin. Plasma was stored frozen at -80ºC until analysis. 200µl plasma add 800µl acetonitrile to protein precipitation. After centrifuge, liquid supernatants were transferred to a new tube and diluted with 60:40 (v:v) water:acetonitrile, and injected on the liquid chromatography-tandem accurate mass spectrometry system. Reversed phase liquid chromatography coupled to positive ion electrospray tandem quadrupole mass spectrometry was conducted (1). In brief, Agilent C18, 1.8µm, 2.1×100mm analytical column was used. Mobile phases A and B were 0.1% formic acid and acetonitrile. The flow rate was 0.5 mL/min. The gradient program included 70:30 mobile phase A:B 0.1minutes and 60:40 mobile phase A:B 5 minutes. 15:85 mobile phase A:B wash the column for 6 minutes, and then 70:30 mobile phase A:B was used to re-equilibrate for 8 minutes. A Sciex QTrap 6500 tandem quadrupole mass spectrometer equipped with electron spray ionization was operated in positive mode. Multiple reaction monitoring for nirmatrelvir (PF07321332) detection was set 500.5>110.3. Peak areas of the analytes and standards (PF-07321332, MedChemExpress) were performed by SCIEX OS software version 1.5. The calibration curve was constructed by using peak area ratios of the calibration samples and applying weighted ($1/X^2$) linear least squares regression analysis. The validation range was 10-10,000 ng/mL.

Reference

1. Singh RSP, Toussi SS, Hackman F, Chan PL, Rao R, Allen R, et al. Innovative Randomized phase I study and dosing regimen selection to accelerate and inform pivotal COVID-19 trial of nirmatrelvir. *Clin Pharmacol Ther* 2022;10.1002/cpt.2603. doi:10.1002/cpt.2603

Demographic and clinical characteristics of two groups of the hemodialysis patients

Table 1. Demographic and clinical characteristics of two groups of the hemodialysis patients

	Treatment with Nirmatrelvir/Ritonavir (N=14)	Treatment without Nirmatrelvir/Ritonavir (N=35)	*P value*
General characteristics			
Age (years)	66.86±10.70	57.77±13.76	0.032
Female, n (%)	7(50.0%)	6(17.1%)	0.019
BMI（kg/m2）	23.47±5.03	23.52±4.01	0.973
Hemodialysis vintage (months)	44.2±65.3	48.0±51.5	0.829
Complications, n (%)			
Diabetes	6(42.9%)	10(28.6%)	0.335
Coronary heart disease	5(35.7%)	10(28.6%)	0.624
pneumonia	8(57.1%)	10(28.6%)	0.061
Laboratory results			
WBC ($\times 10^9$/L)	4.63±1.86	5.45±1.91	0.187
Lymphocyte ($\times 10^9$/L)	0.66±0.19	0.90±0.47	0.080
Hemoglobin (g/L)	95.0±20.3	91.8±17.8	0.598
Platelet ($\times 10^9$/L)	149.4±56.9	165.2±65.1	0.436
CRP (mg/L)	24.9±42.9	21.5±22.3	0.725
IL-6(ng/ml)	23.7±17.8	27.4±21.9	0.611
Albumin (g/L)	36.3±3.8	36.8±4.5	0.691
Alt (U/L)	36.0±54.3	11.3±9.5	0.113
Ast(U/L)	22.9±8.7	15.3±6.6	0.002
Total bilirubin (μmol/L)	8.6±3.5	9.6±2.9	0.302
D-Dimer (μg/ml)	0.68±0.66	0.76±0.95	0.771
